# Grain-rich diets altered the colonic fermentation and mucosa-associated bacterial communities and induced mucosal injuries in goats

**DOI:** 10.1038/srep20329

**Published:** 2016-02-04

**Authors:** Huimin Ye, Junhua Liu, Panfei Feng, Weiyun Zhu, Shengyong Mao

**Affiliations:** 1 College of Animal Science and Technology, Nanjing Agricultural University, Nanjing 210095, China

## Abstract

Remarkably little information is available about the impact of high-grain (HG) feeding on colonic mucosa-associated bacteria and mucosal morphology. In the present study, 12 male goats were randomly assigned to either a hay diet (n = 6) or an HG diet (65% grain; n = 6) to characterise the changes in the composition of the bacterial community in colonic mucosa and the mucosal morphology of the colon. The results showed that HG feeding decreased the colonic pH and increased the concentrations of total short chain fatty acids and lipopolysaccharides in colonic digesta. The principal coordinate analysis results showed that the HG diet altered the colonic mucosal bacterial communities, with an increase in the abundance of genus *Blautia* and a decrease in the abundance of genera *Bacillus, Enterococcus,* and *Lactococcus*. The HG-fed goats showed sloughing of the surface layer epithelium, intercellular tight junction erosion, cell mitochondrial damage, and upregulation of the relative mRNA expression of IL-2 and IFN-γ in colonic mucosa. Collectively, our data indicate that HG feeding induced changes in colonic mucosal morphology and cytokines expression that might be caused by excessive fermentation and dramatic shifts in the bacterial populations in the colon.

Meat goats are often fed relatively high-grain (HG) diets to achieve maximum production^1^. Usually, feeding HG diets tends to increase meat production. However, feeding diets high in readily fermentable carbohydrates increases the probability of developing subacute ruminal acidosis (SARA) and decreases the long-term productive performance of goats^2^. During SARA, the rate of ruminal short-chain fatty acid (SCFA) production exceeds SCFA absorption and results in an unhealthy depression of ruminal pH, further causing a shift in the rumen microbiota^3^,^4^. Events that occur in the rumen during SARA are mirrored in the hindgut. A previous study revealed that an increase in intestinal carbohydrate fermentation typically occurs concurrently with HG feeding and may lead to increased concentrations of SCFA and lipopolysaccharide (LPS), as well as reduced pH in the colons of goats^4^. This reduction might indicate that colonic fermentation characteristics are altered by HG feeding. This alteration in fermentation characteristics may affect the composition of the colonic bacterial communities due to the susceptibility of some bacteria to low pH^5^. Indeed, Metzler-Zebeli *et al.* (2013) reported that HG feeding had an impact on colonic digesta-associated bacterial populations in goats^4^. However, similar information regarding colonic epithelial bacteria is incomplete compared to knowledge of the bacterial community in the colonic luminal content of goats fed HG diets.

The colonic epithelium is inhabited by mucosa-associated bacteria and represents a physical and immunological barrier^6^. Thus, maintenance of the structural and functional integrity of the mucosal epithelium is extremely important. Previous studies have found that HG feeding induced diarrhoea, frothy faeces, and increased particle size in faeces^5^,^7^. In addition, mucin casts have been observed in the faeces of dairy cattle during SARA, indicating that HG feeding may lead to hindgut mucosal injury in ruminants^5^,^7^. However, there is little information regarding changes in the histological structure and ultrastructure of the colonic mucosa of ruminants during HG feeding.

Herein, we hypothesised that HG diet feeding might cause changes in colonic fermentation characteristics and the mucosal-associated bacterial community, and that these changes might induce colonic mucosal injury in goats. Therefore, the objective of the present study was to investigate the changes in colonic fermentation characteristics, mucosal bacterial composition, and mucosal morphology during HG feeding.

## Results

The goats were healthy throughout the experiment. HG feeding had no significant effect on heartbeat, rectal temperature, or ruminal contraction.

### Colonic digesta pH and concentrations of SCFA, lactate, and LPS

The dietary effects on colonic digesta variables are presented in Table 1. Compared with the hay diet, the HG diet caused a decrease in the pH of colonic digesta (7.44 ± 0.15 vs. 6.74 ± 0.10; *P* = 0.003). The HG diet increased the concentrations of lactate and free LPS by 75% (*P* < 0.001) and 225% (*P* = 0.011), respectively. Compared with the hay diet, the HG diet increased the concentrations of acetate (25.65 ± 1.68 vs. 60.11 ± 2.59 μmol/g; *P* < 0.001), propionate (5.17 ± 0.38 vs. 12.07 ± 0.81 μmol/g; *P* < 0.001), isobutyrate (0.86 ± 0.06 vs. 1.63 ± 0.11 μmol/g; *P* < 0.001), butyrate (1.31 ± 0.14 vs. 5.14 ± 0.82 μmol/g; *P* = 0.005), isovalerate (0.77 ± 0.09 vs. 1.50 ± 0.16 μmol/g; *P* = 0.003), valerate (0.49 ± 0.04 vs. 1.12 ± 0.14 μmol/g; *P* = 0.006), and total SCFA (34.25 ± 2.27 vs. 81.56 ± 3.71 μmol/g; *P* < 0.001) in the colonic digesta (Table 1).

### Bacterial composition determined by MiSeq sequencing

Bacterial communities of colonic mucosa were determined by Illumina MiSeq sequencing of 16S rRNA genes and resulted in the recovery of 871,778 effective sequences with a read length ≥200 bp. After quality filtering by QIIME, 818,183 high-quality reads were obtained, accounting for 93.85% of the raw reads. Total sequences were assigned to 28 phyla from the colonic mucosa of all the goats. The number of phyla detected in both groups was 24. At the genus level, 296 and 307 taxa were detected in the colonic mucosa of the hay diet-fed and HG diet-fed groups, respectively.

At the phylum level, bacteria belonging to the phylum Firmicutes were the most dominant, accounting for 67.34% of total sequences, followed by bacteria from the phyla Bacteroidetes (13.24%), Proteobacteria (10.92%), Spirochaetes (2.87%), and Actinobacteria (1.42%) (Supplemental Fig. S1). Together with the unclassified bacteria (1.97%), they accounted for more than 95% of the total sequences. The rare phyla included Nitrospirae, Planctomycetes, Candidate_division_SR1, Armatimonadetes, Candidate_division_OD1, Candidate_division_BRC1, and Chlorobi (≤0.01%). At the genus level, the dominant taxa were composed of *Lactococcus* (24.03%), unclassified Ruminococcaceae (17.64%), *Pseudomonas* (5.24%), *Enterococcus* (3.26%) and unclassified Rikenellaceae (3.11%). For visualisation, the top fifty bacterial taxa are presented in a heat map (Supplemental Fig. S2).

### Effect of HG diet on the diversity and composition of colonic mucosal bacteria

**Figure 1:**
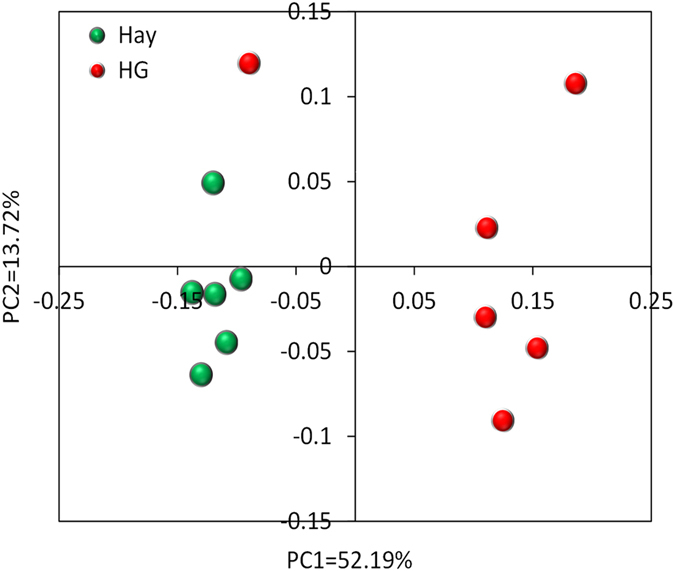
Principal coordinate analysis (PCoA) of bacterial communities in the colonic mucosal samples.

As shown in Supplemental Fig. S3, the rarefaction curves approached a plateau, suggesting that further sequencing would not result in the increase of operational taxonomic units (OTUs) in each group. The numbers of OTUs, chao1, ACE, and Shannon index are listed in Table 2. At the 0.03 dissimilarity level, the values of ACE and chao 1 were significantly decreased by the HG diet (*P* = 0.001),whereas no difference (*P* = 0.513) was detected in the Shannon index between the hay and HG groups. Principal coordinate analysis (PCoA) plots based on unweighted UniFrac distance metrics showed a separation between the hay and HG groups using PC1 and PC2 (52.19% and 13.72%, respectively, of the explained variance) (Fig. 1). In addition, the bacterial communities of the colonic mucosa [analysis of molecular variance (AMOVA): Fs = 6.718, *P* = 0.003] were significantly affected by diet. Among all the phyla detected, the abundance of phylum Bacteroidetes was higher (*P* = 0.020) in the HG diet compared with the hay diet (Supplemental Fig. S1). At the genus level, the abundance of *Blautia* was 1.21% and 0.07% in the HG and hay groups, respectively, indicating that the HG diet significantly increased the abundance of bacteria belonging to the genus *Blautia* (*P* = 0.029) (Table 3). In contrast, there was a significant decrease in bacteria from the *Bacillus* (*P* = 0.045), *Enterococcus* (*P* = 0.021), *Lactococcus* (*P* = 0.033), unclassified Peptostreptococcaceae (*P* = 0.002), and *Psychrobacter* (*P* = 0.013) taxa in the HG group compared with the hay group.

At the OTU level, the study found 2,203 OTUs across all samples, 501 exclusively in the hay group and 498 in the HG group (Supplemental Fig. S4). Of them, one OTU, OTU-1454, classified in the genus *Lactococcus* was the most dominant among the dominant OTUs (representing ≥2% of all sequences in at least one group in all samples) (Supplemental Fig. S5). Among the 2,203 OTUs detected in the present study, 7 dominant OTUs (relative abundance ≥1% in at least one group) were affected significantly by the diet (Supplemental Fig. S5). Among the OTUs affected, the dominant species, including OTU-1454 (S: *Lactococcus piscium*) (*P* = 0.032), OTU-601 (G: *Enterococcus*) (*P* = 0.02), OTU-668 (S: *Lactococcus lactis* subsp) (*P* = 0.042), OTU-2403 (G: *Pseudomonas*) (*P* = 0.019) and OTU-638 (F: Peptostreptococcaceae) (*P* < 0.001) were increased in percentage in the hay diet-fed group compared with the HG-fed group, while the abundance of OTU-2527 (F: Ruminococcaceae) (*P* = 0.005) and OTU-1571 (F: Ruminococcaceae) (*P* = 0.022) were reduced.

### Metagenomic metabolic functions of colonic mucosal bacteria

**Figure 2:**
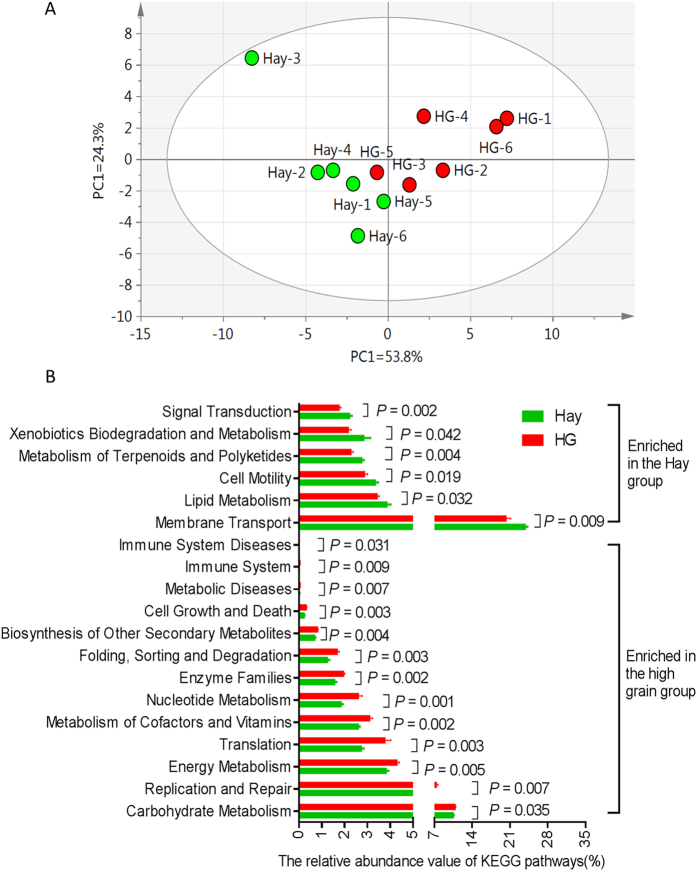
Functional diversity of the bacterial microbiota of colonic mucosa. (**A**) Principal component analysis of KEGG pathways encoded in the microbiota of colonic mucosa in hay and HG group. (**B**) Influence of HG feeding on the abundance of KEGG pathways of colonic mucosal microbiota of goats. Only the KEGG pathways that were significantly affected in percentage by diet are presented.

Using PICRUSt as a predictive exploratory tool, we found that a total of 38 gene families were identified in all samples (Supplemental Fig. S6). By performing a principal component analysis (PCA) on the relative abundance values of the KEGG pathways from the mucosal-associated bacteria of the goats, we found that the hay and HG samples clustered differently (Fig. 2A). In the 38 gene families, the majority of the genes that were obtained corresponded to membrane transport (23.87% in the mucosal-associated bacteria of the hay group and 20.31% in the HG group), carbohydrate metabolism (10.54% in hay, 10.88% in HG), amino acid metabolism (8.54% in hay, 8.89% in HG), and replication and repair (5.88% in hay, 7.40% in HG) (Supplemental Fig. S5). Of the 38 gene families, 19 were significantly differentially abundant between the Hay and HG groups (*P* < 0.05) (Fig. 2B). Compared with Hay-fed goats, HG-fed goats had higher relative abundance of gene families carbohydrate metabolism (*P* = 0.0353), replication and repair (*P* = 0.007), energy metabolism (*P* = 0.005), translation (*P* = 0.003), metabolism of cofactors and vitamins (*P* = 0.002), nucleotide metabolism (*P* = 0.001), enzyme families (*P* = 0.002), folding, sorting and degradation (*P* = 0.003), biosynthesis of other secondary metabolites (*P* = 0.004), cell growth and death (*P* = 0.003), metabolic diseases (*P* = 0.007), immune system (*P* = 0.009), and immune system diseases (*P* = 0.031), while lower relative abundce of gene families membrane transport, lipid metabolism (*P* = 0.032), cell motility (*P* = 0.019), metabolism of terpenoids and polyketides (*P* = 0.004), xenobiotics biodegradation and metabolism (*P* = 0.042), and signal transduction (*P* = 0.002).

### Morphology and ultrastructure of the colon epithelium

**Figure 3:**
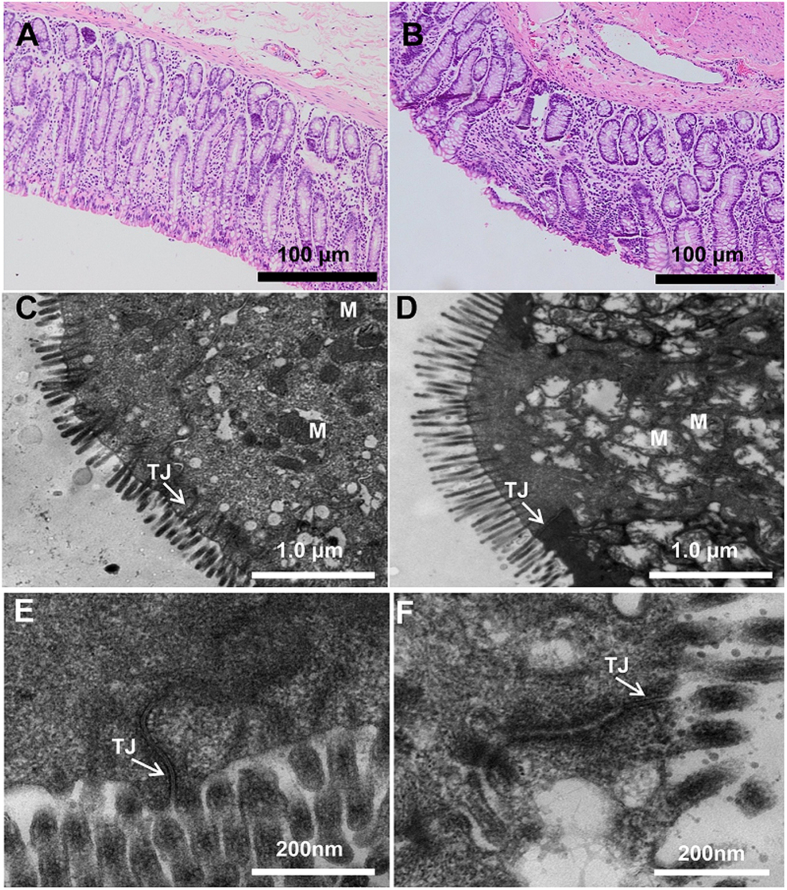
Histology of colon tissue, comparing hay-fed and HG-fed goats. Light microscopy cross-section of colon tissue from a representative hay-fed goat ((**A**) scale bar = 100 μm) or HG-fed goat ((**B**) scale bar = 100 μm). Comparison of colonic epithelial ultrastructure between hay-fed goat ((**C**) scale bar = 1 μm) and HG-fed goat ((**D**) scale bar = 1 μm). Colonic epithelial ultrastructure of junctional complexes in representative hay-fed goat ((**E**) scale bar = 200 nm) and HG-fed goat ((**F**) scale bar = 200 nm). M: mitochondria; TJ: tight junction.

Representative light micrographs of the cross-sections of the colonic epithelia of the hay-fed goats show that the crypt orifices were circular in outline and the intercryptal surface was partially covered by an irregular layer of mucus (Fig. 3A). In contrast, the HG-fed goats exhibited sloughing of the epithelial surface (Fig. 3B). The colonic lesion score of HG-fed goats was significantly higher than that of hay-fed goats (4.56 ± 0.59 vs. 2.41 ± 0.40). The ultrastructure of the colon epithelium is shown in Fig. 3C–F. The hay-fed goats had clear and organised microvillus clusters (Fig. 3C), while the microvilli of the HG-fed goats were sparse and irregular (Fig. 3D). In the hay-fed goats, the integrity of the cell organelles in the microvilli was normal and the tight junction band was clearly visible (Fig. 3E). In the HG-fed goats, numerous vacuoles appeared in the cell layers. Mitochondrial swelling and intercellular tight junction erosion were also apparent (Fig. 3F).

### Gene expression in the colonic mucosa

**Figure 4:**
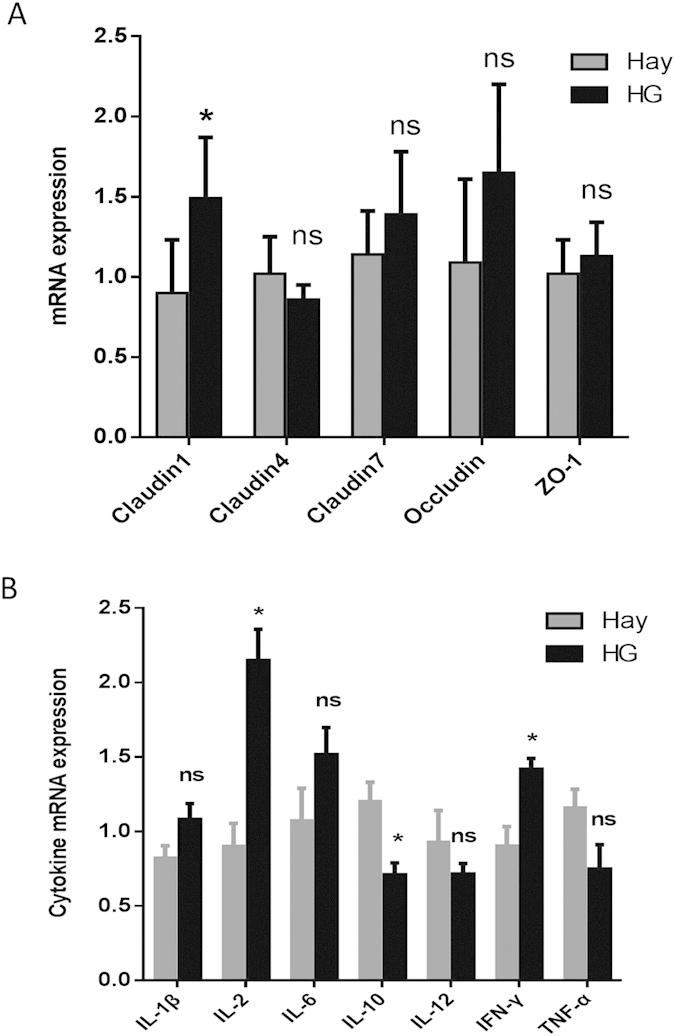
Effects of HG feeding on the relative mRNA expression of tight junction protein and cytokines in the colon of goats.

The relative mRNA expression of cytokines and tight junction protein was determined by real-time quantitative polymerase chain reaction (RT-qPCR). As shown in Fig. 4A, the mRNA expression level of IL-2 and IFN-γ was up-regulated by 137% (*P* = 0.001) and 57.7% (*P* = 0.005) during HG diet feeding, respectively. In contrast, the expression level of IL-10 was down-regulated significantly in the goats fed the HG diet. There were no significant differences in the mRNA expression of IL-1β (*P* = 0.101), IL-6 (*P* = 0.169), IL-12 (*P* = 0.383), or TNF-α (*P* = 0.087) between the hay and HG groups. Overall, compared with the hay-fed goats, the HG-fed goats had higher levels of claudin-1 (*P* = 0.001) mRNA expression, and there were no significant differences (*P* > 0.05) in the mRNA expression of claudin-4, claudin-7, occludin, or ZO-1 between the two groups (Fig. 4B).

### Correlation analysis

**Figure 5:**
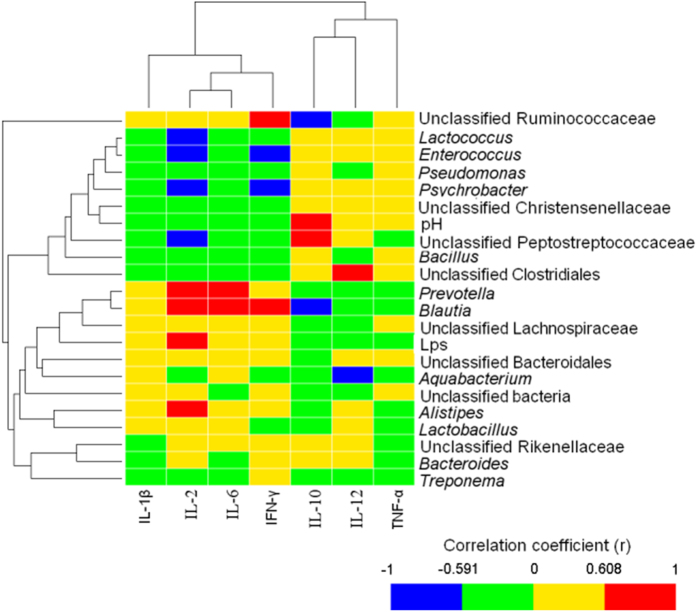
Correlation analyses between the colonic pH, colonic LPS levels and mucosa-associated microbiota (at the genus level) and the levels of mucosal inflammatory cytokines expression. Only the predominant bacterial genera (relative abundance ≥1% in at least one sample) for which abundance was significantly associated with inflammatory cytokines expression are presented; Cells are colored based on Pearson correlation coefficient between the colonic pH, colonic LPS levels, mucosa-associated microbiota and the levels of inflammatory cytokines expression in colonic mucosa. The red color represents a significant positive correlation (*P* < 0.05), the blue color represents a significant negative correlation (*P* < 0.05) and the green and yellow shows that the correlation was not significant (*P* > 0.05).

The correlative relationships among mucosal inflammatory cytokine expression and colonic digesta pH, LPS concentration, and mucosa-associated predominant bacterial populations (relative abundance ≥1% in at least one sample) were evaluated in this study (Fig. 5). The results showed that colonic pH correlated negatively with the relative mRNA expression of IL-1β (r = −0.591; *P* = 0.047) and IFN-γ (r = −0.611; *P* = 0.035), and positively with the mRNA expression of IL-10 (r = 0.767; *P* = 0.005) in the mucosa. In addition, the abundance of seven taxa [four negative: *Lactococcus* (r = −0.594; *P* = 0.046), *Enterococcus* (r = 0.636; *P* = 0.03), unclassified Peptostreptococcaceae (r = −0.601; *P* = 0.043), and *Psychrobacter* (r = −0.629; *P* = 0.032); three positive: *Prevotella* (r = 0.734; *P* = 0.009)*, Alistipes* (r = 0.657; *P* = 0.023) and *Blautia* (r = 0.762; *P* = 0.006)] was correlated with the increased relative mRNA expression of IL-2. The abundance of two genera [*Prevotella* (r = 0.671; *P* = 0.020) and *Blautia* (r = 0.699; *P* = 0.014)] was positively associated with the relative mRNA expression of IL-6, and the abundance of three taxa [two negative: unclassified Ruminococcaceae (r = −0.622; *P* = 0.035) and *Blautia* (r = −0.650; *P* = 0.026), and one positive: unclassified Peptostreptococcaceae (r = 0.629; *P* = 0.031)] was correlated with the relative mRNA expression of IL-10. The proportion of two taxa [one negative: *Aquabacterium* (r = −0.685; *P* = 0.017) and one positive: unclassified Clostridiales (r = 0.608; *P* = 0.039)] was linked to the mRNA expression of IL-12, and four taxa [two positive: unclassified Ruminococcaceae (r = 0.650; *P* = 0.025) and *Blautia* (r = 0.671; *P* = 0.020), and two negative: *Enterococcus* (r = −0.643; *P* = 0.028) and *Psychrobacter* (r = −0.594; *P* = 0.046)] were correlated with the relative mRNA expression of IFN-γ. No significant correlations (*P* < 0.05) were found between the abundance of mucosa-associated predominant bacterial populations and the relative mRNA expression of TNF-α.

## Discussion

Over the last few decades, diets rich in highly fermentable carbohydrates have commonly been fed to achieve a high level of milk production or body weight gain in ruminants^8^,^9^,^10^. An HG diet results in an increased amount of fermentable carbohydrates passing to the hindgut, thus increasing fermentation in the hindgut^5^,^7^. In the current study, HG diets decreased the colonic pH and increased the total SCFA concentration in the colon. These results are consistent with the reports mentioned earlier^5^,^7^, and they suggest that HG feeding results in excessive fermentation in the hindgut of goats.

Compared to the advanced understanding of bacteria associated with the ruminal and cecal epithelium and in the colonic digesta and its adaptation to different dietary regimens^3^,^4^,^11^,^12^, shifts in the colon mucosal bacterial community of goats in response to dietary changes are not as well characterised. In the present study, we investigated the adaptive response of the colon mucosal bacteria to HG feeding using high-throughput sequencing methods. In line with previous findings in bacterial communities in the colonic mucosa of calves^13^ and in the ruminal epithelia of goats^11^, our results revealed that the Firmicutes and Bacteroidetes are the predominant phyla in colonic mucosa across all samples, accounting for over 80% of the total sequences. Bacteria belonging to the Firmicutes and Bacteroidetes phyla are known for a fermentative metabolism and degradation of carbon sources, oligosaccharides^14^,^15^, protein and amino acid^16^. The high abundance of Firmicutes and Bacteroidetes in colonic mucosal-associated bacteria suggest that they have a role in the utilisation of carbohydrates, protein and amino acid. Nevertheless, among the dominant phyla in the colon mucosa, the phylum Firmicutes contained the most bacteria with 67.34% of total sequences, followed by the phyla Bacteroidetes (13.24%) and Proteobacteria (10.92%), compared to the dominant bacteria (Firmicutes: 50% of total sequences) in the colon tissue of pre-weaned calves^13^. The different percentage of dominant phyla in colon mucosa may be explained by the different diets and hosts used in the studies.

In the present study, we observed that HG feeding decreased the richness of colonic mucosal bacteria, which was confirmed by richness estimates based on chao1 and ACE. The results of unweighted UniFrac PCoA and AMOVA further revealed the bacterial community difference between the two groups, indicating that HG feeding altered the composition of the mucosa-associated bacterial community. This alteration may be due to several reasons. First, the strong effect of a decrease in colonic pH might contribute to the changes in the colonic mucosal bacterial community. There is sufficient information available regarding the susceptibility of rumen and hindgut bacteria to low pH^3^,^11^,^12^. In the present study, the lower colonic pH might decrease the richness of some colonic mucosal bacteria due to their susceptibility to low pH, while increased abundance of some low pH-tolerant colonic mucosal bacteria. Second, variations in the content of dietary components between the two groups might also have resulted in the differences in colonic bacterial response. During HG feeding, dietary-derived nutrients such as starch are likely to be used by mucosa-associated bacteria during HG feeding, thus resulting in an increase in the percentage of polysaccharide-degrading bacteria such as *Blautia*^17^. Third, and perhaps most important, colonic mucosal injury induced by HG feeding may affect the colonic mucosal bacterial community by increasing the availability of attachment sites for opportunistic bacteria.

At the genus level, the relative abundances of *Bacillus, Enterococcus, Lactococcus, Psychrobacter* and *Pseudomonas* decreased in HG-fed goats. Some strains of *Lactococcus,* the lactate producers, are important members of the endogenous bacteria that play an anti-inflammatory role in humans^18^. The decrease in the abundance of *Lactococcus* in the HG-fed group in this study indicates that an HG diet may be associated with the local inflammation in colon. Indeed, our data revealed that the goats fed the HG diet showed a significantly lower abundance of *L. piscium* (OTU-1454) and *L. lactis* subsp (OTU-668) (Supplemental Fig. S5). As the probiotic effect of *L. pisciu*m and *L. lactis* on human health has been extensively documented^19^,^20^. The decrease in the abundance of *L. pisciu*m and *L. lactis* in the HG group further demonstrated that feeding an HG diet may have negative effects on mucosal health. In the present study, a possible reason for the decrease in the proportion of *Psychrobacter* and *Pseudomonas* (both gram-negative bacteria) in the HG-fed group may be related to the lower pH of the colonic digesta that resulted from the HG feeding, as gram-negative populations are known to be sensitive to low pH^21^.

It has been reported that the gut bacteria make an important functional contribution in maintaining the health of the host^22^,^23^. In the present study, PICRUSt was used to determine the potential functions of mucosa-associated bacteria in colons across all samples. The distinct difference in predicted functional pathways between the hay and HG groups indicates that alterations of bacterial communities are also likely to alter the functional capability of the colonic mucosa-associated bacterial community. Our data showed that compared with the control group, the genes responsible for carbohydrate metabolism were up-regulated in the HG group, while there was a decrease in the genes involved in lipid metabolism. This coordinated change in gene expression suggests a selective shift in metabolic pathways favouring the use of carbohydrates as fuel to sustain energy expenditure for the mucosa-associated bacteria. In addition, an increased percentage of bacterial inferred genes associated with immune system diseases were observed in the HG group, suggesting the possibility that long-term patterns of HG diet consumption may have detrimental effects on host immune function. In the present study, a relative increase in genes associated with energy metabolism pathways was also present in the HG group, indicating that the mucosal microbiomes associated with HG feeding may have an increased capacity for energy harvest. In general, these predicted data indicates that long-term HG diet feeding may alter the bacterial function of the colonic mucosal microbiome in goats. However, it must be noted that PICRUSt’s well-validated predictive approach cannot confirm with absolute certainty the functional capabilities of the metagenome. Thus, true metagenomic shotgun sequencing is needed to provide greater power in order to examine the effects of feeding HG diets on the function of mucosa-associated bacteria in the colon of goats.

The changes in gastrointestinal fermentation and the mucosal bacterial community may further affect the histological integrity and function of the gastrointestinal epithelium. Previous studies have revealed that feeding HG diets to ruminants causes a high risk of damage to the histological integrity and functions of the ruminal epithelium in dairy cows and goats^24^,^25^,^26^. However, there are very few reports on the influence of an HG diet on colonic epithelial structure and function. The present study showed that feeding an HG diet to goats for seven weeks resulted in sloughing of the surface layer epithelium, intercellular tight junction erosion and cell mitochondrial damage. In addition, we observed that HG feeding up-regulated the proinflammatory cytokines (IL-2 and IFN-γ) and decreased the anti-inflammatory cytokine (IL-10) compared with the control. There are many possible explanations for the mucosal injury in the HG group. First, excessive carbohydrates in the colons of HG-fed goats may increase the osmotic pressure of digesta. High osmotic pressure is undoubtedly a threat to the epithelial barrier and may increase the permeability of the colonic epithelium^5^. Second, luminal acidity is one of the most important factors in determining the status of the epithelial barrier. A previous study reported that acetic acid (0.1 M) showed a time- and pH-dependent ability to damage the colonic epithelium in pigs and that its mechanism is probably related to the ability of lower environmental pH to stimulate the SCFA entering into the non-glandular mucosal cells, acidifying the cell and damaging chloride-dependent Na transport, which results in cell necrosis^27^. In the present study, the level of acetate and other SCFA components in the colonic digesta was markedly higher in HG-fed goats, which might increase localisation epithelial necrosis.

While this study reveals the detrimental effects of an HG diet on the integrity of the intestinal epithelium, the principle mechanisms responsible for triggering a local immune response during HG feeding are unknown. In the current study, correlation analysis revealed that there is a relationship between pH and some Inflammatory cytokines, such as IL-1β, IFN-γ, and IL-10. These results indicate that the lower pH may partly contribute to the changes in colonic cytokines expression in the HG group. Indeed, the influence of lower pH on inflammation in the colon is well documented^28^. It is well known that SCFAs have high lipid solubility. When environmental pH declines to the threshold, the SCFA can enter into the non-glandular mucosal cells, acidifying the cell and damaging chloride-dependent Na transport, which results in cell necrosis^29^.

It should be stressed that it is not only lower pH that can affect mucosal cytokines expression; there are also some pathogenic and opportunistic pathogenic bacteria that can induce inflammation. Previous studies have revealed that intestinal mucosal tissue damage in colitis cases might result mostly from the production of inflammatory cytokines in response to commensal bacteria^30^,^31^. In the present study, the correlation analysis of the composition of mucosa-associated bacteria and the inflammatory parameters showed that the increase in the pro-inflammatory cytokines correlated with changes in some mucosal bacteria of goats. In particular, the increased IL-2 and IFN-γ mRNA expression observed in the goats fed the HG diet, were linked to enrichments in *Blautia* in the HG group. As mentioned earlier, some of the *Blautia* phylotypes are known to have deleterious mucosal injury^32^,^33^. In addition, the correlation analysis showed that the genus *Alistipes*, which is directly connected to elevated inflammation levels in the human gut and is known to have deleterious mucosal injury effects^34^,^35^, is positively correlated with the relative mRNA expression of IL-2 and IL-6. This implies that the inflammation that occurs in the hindgut mucosal epithelium may also partly be due to the change in the abundance of genus *Alistipes*.

However, it has not been concluded whether variations in colonic pH and the mucosal bacterial community are the factors that contribute to the initial development of local inflammation in the colonic epithelium during HG diet feeding. Indeed, a very recent study regarding the expression of genes and proteins with key protection functions in the rumen epithelia of goats fed low-grain and HG diets revealed a mismatch in gene and protein expression in response to grain level^36^, indicating that the upregulation in the expression of cytokine genes might not result in increased expression of the corresponding proteins. Thus, future studies are needed to determine whether differences in the colonic mucosal bacterial communities of hay and HG groups are associated with differences in the protein expression of cytokines.

There has been remarkably little information regarding the consequences of damage to the colonic epithelium on the function of the colon and animal performance. However, recent studies on the influence of rumen acidosis on rumen function have highlighted the possibility that injury to the colonic epithelium has a potential impact on colon function. Previous studies have revealed that rumen acidosis may increase the permeability of the rumen epithelium^37^,^38^, and increased permeability may increase the uptake of toxic compounds such as histamine generated in the rumen into the body and the uptake of SCFAs^39^. In addition, chronic inflammation caused by damage to the rumen epithelium may stimulate the release of cytokines and trigger an immune response^38^, the latter of which may decrease feed intake and diet digestibility^40^. Further, recent studies have revealed that hindgut acidosis decreased diet digestibility and milk fat percentage in cattle, and that the decreased diet digestibility likely resulted from increased bulk in the digestive tract or from increased digesta passage rate, reducing exposure of the digesta to intestinal enzymes and epithelial absorptive surfaces^5^,^41^. Permeability of the colonic epithelium and diet digestibility in the colon were not measured in the current study. However, Emmanuel *et al.*^42^ showed that low pH in the presence of LPS increased the permeability of colon epithelia in cattle. Therefore, these findings indicate that the damage to the colonic epithelium due to HG feeding may have a potential adverse impact on colon function and animal performance.

In conclusion, this study showed that HG feeding caused decreased colonic pH in goats and increased concentrations of total colonic SCFA, lactic acid, and LPS, indicating increased fermentation and the production of free LPS in the large intestine. Data from this study also demonstrated that HG diet feeding led to the changes in the colonic mucosal bacterial community, with an increase in the abundance of the genus *Blautia* and a decrease in the abundance of genera *Bacillus, Enterococcus* and *Lactococcus*. Microscopic examinations of the colonic mucosa revealed that the HG diet resulted in mucosal epithelial injury, and RT-PCR revealed that HG caused the up-regulated expression of IL-2 and IFN-γ in the colonic mucosa. Correlation analysis revealed that the alteration in pH and mucosa-associated bacteria during HG feeding might partly contribute to changes in expression of colonic mucosal cytokines. Overall, these results provide a comprehensive picture of the bacterial structure of the colon during HG feeding and help to further elucidate the etiology of hindgut disorders in ruminants.

## Materials and Methods

### Ethical approval

Our animal experiment was approved by the Animal Experiment Committee of Nanjing Agricultural University, in accordance with the Regulations for the Administration of Affairs Concerning Experimental Animals (The State Science and Technology Commission of China, 1988). All experiments were performed in accordance with the approved guidelines and regulations.

### Animals and experimental procedures

This animal experiment was carried out at the experimental station of Nanjing Agricultural University in Jiangsu Province, China. Twelve, eight-month-old male goats were used in this study. At the commencement of the experiment, the goats had free access to hay and water for two weeks during the adaption period. After adaption, the goats were randomly allocated to groups fed either hay (hay; 0% concentrate; n = 6), or an HG diet containing 75% concentrate and 25% hay (HG; 75% concentrate; n = 6). There were no significant differences in the body weights of the goats in the hay and HG groups (28.10 ± 1.23 vs. 26.47 ± 0.83 kg) at the commencement of the feeding trial. Adaptation to the HG diet was carried out over 5 d with progressively increasing amounts of grain up to 65%. Water was available ad libitum. The diet (900 g dry matter per animal per day) was offered in equal amounts at 0800 h and 1700 h daily for seven weeks. The metabolic energy intake of the goats in the hay group (30 kg body weight; 8.31 MJ/kg dry matter) was slightly above the requirement for maintenance, and that of the goats in the HG group (30 kg body weight; 11.31 MJ/kg dry matter) permitted a growth rate of 200 g/day according to guidelines on the nutrient requirements of goats (NY/Y816-2004; Ministry of Agriculture of China, 2004). The components and nutrient compositions of the diets are shown in Supplemental Table S1. The health statuses of the goats were monitored throughout the experiment (heartbeat, rectal temperature, and ruminal contractions were measured every week).

### Sample collection

The goats were slaughtered four hours after the morning feeding on day 50. The abdominal cavity was opened and the entire gastrointestinal tract was removed. A representative sample of colon digesta was collected to determine the pH value, then the whole colon digesta were homogenized and divided into two portions. The first portion of each of these samples (10 g wet weight) was mixed thoroughly with a double amount of distilled water. The mixtures were immediately centrifuged at 2,000 × g and the supernatants were stored at −20 °C until they were analyzed for SCFA and lactic acid^43^. The second portion of each of the colon digesta samples (10 g wet weight) was mixed with an equal amount of endotoxin-free water and the mixtures were immediately centrifuged at 13,000 × g for 40 minutes at 4 °C, and the supernatant was gently pushed through a 0.22-μm LPS-free filter. The filtrate was stored in a sterile glass tube and heated at 100 °C for 30 min. The glass tubes were then kept at −20 °C for LPS analysis. Within five minutes after slaughter, a segment of the colon tissue was collected and immediately washed three times in ice-cold phosphate-buffered saline. The samples were divided into three portions. The first portion of the tissue sample was cut into smaller pieces of approximately 0.5 × 0.5 cm and was immediately frozen in liquid nitrogen for RNA extraction. The second portion of tissue sample was cut into approximately 2 × 2 cm and scraped from the underlying tissue using a germ-free glass slide, and was immediately transferred into liquid N, and then stored at −80 °C until it was required for DNA extraction. The last portion was immediately fixed in 4% paraformaldehyde (Sigma, St. Louis, MO, USA) and 2.5% glutaraldehyde for histomorphometric microscopy analysis.

### Physiological parameters measurements

Colonic digesta pH was immediately determined with a portable pH-meter (HI 9024C; HANNA Instruments, Woonsocket, RI, USA). The pH meter was calibrated with a standard pH solution before measuring pH in colonic digesta. The SCFA were analysed by gas chromatography (GC-14B; Shimadzu, Kyoto, Japan; capillary column: 30 m × 0.32 mm × 0.25 μm; temperature of the column 110 °C, temperature of the injector 180 °C, temperature of the detector 180 °C)^43^. Lactate levels in the colonic digesta were determined by using a Lactate Assay Kit (Nanjing Jiancheng Bioengineering Institute, Nanjing, China). Free LPS in the colonic content was measured via a Chromogenic End-point Tachypleus Amebocyte Lysate Assay Kit (Xiamen Horseshoe Crab Reagent Manufactory, Ximen, China) with a minimum detection limit of 0.1 EU/mL. Pretreated supernatant was diluted until their LPS concentrations were in the range of 0.1 to 1 EU/mL relative to the reference endotoxin.

### Histological measurements

The colonic tissues were opened lengthwise, embedded in paraffin, sectioned (6 μm), and stained with hematoxylin and eosin (H&E). The microscopist was blinded to treatment conditions during the histomorphometric analysis. Measurements of lesions were made using the 40 × objective lens. Three slides per goats were prepared and two images were captured per slides for a total of 36 replicates per measurement per group. Image Pro Plus software (Media Cybernetics, Bethesda, MD, USA) was used to measure predefined criteria previously described by Steel *et al.*^44^. In brief, a score of one indicated nil to minor lesions, a score of five indicated minor lesions with mucusa sloughing, and a score of nine indicated severe deep lesions with large amounts of mucosa sloughing. The tissues were fixed with 2.5% glutaraldehyde for at least 24 h, postfixed in 1% osmium, and embedded in Epon araldite. Semithin sections (0.25–0.5 μm) were cut with a glass knife and stained with 1% toluidine blue and 1% sodium borate. Ultrathin sections (70–90 nm) were cut and stained with uranyl acetate and lead citrate. The ultrastructures of the colonic epithelia were determined with a transmission electron microscope (H-7650; Hitachi Technologies, Tokyo, Japan). One slides per goats were prepared and three images were captured per slides for a total of 18 replicates per measurement per group.

### Colonic tissue RNA extraction and RT-qPCR

Total RNA was extracted from the colonic mucosal samples with acid using TRIzol (Takara Bio, Otsu, Japan) as described by Chomczynski and Sacchi^45^. The RNA concentration was then quantified using a NanoDrop spectrophotometer (ND-1000UV-Vis; Thermo Fisher Scientific, Madison, WI, USA). The absorption ratio (260/280 nm) of all samples was between 1.8 and 2.0, indicating high RNA purity. Aliquots of RNA samples were subjected to electrophoresis through a 1.4% agarose-formaldehyde gel to verify integrity. The concentration of RNA was adjusted to 1 μg/μl based on optical density and stored at −80 °C. Total RNA (1 μg) was reverse-transcribed using a PrimeScript® RT reagent Kit with gDNA Eraser (Takara Bio, Otsu, Japan), carefully following the manufacturer’s instructions.

The primers for Inflammatory cytokines (IL-1β, IL-2, IL-6, IL-10, IL-12, TNF-α, and IFN-γ), tight junction proteins (claudin-1, claudin-4, claudin-7, occludin, and ZO-1), and GAPDH were described by Liu *et al.*^9^ (Supplemental Table S2). All of the primers were synthesised by Invitrogen Life Technologies (Invitrogen, Shanghai, China). RT-qPCR of the target genes and GAPDH were performed using the ABI 7300 real-time PCR system (Applied Biosystems, Foster, CA, USA) with fluorescence detection of SYBR green dye. Amplification conditions were as follows: 95 °C for 30 s, followed by 40 cycles composed of 5 s at 95 °C and 31 s at 57.5 °C (for GAPDH) or 60 °C (for others). Each sample contained 4 ng cDNA in 2 × SYBRGreen PCR Master Mix (Takara Bio, Otsu, Japan) and 200 nmol/L of each primer in a final volume of 20 μL. All measurements were performed in triplicate. A reverse-transcription-negative blank of each sample and a no-template blank served as negative controls. Gene expression levels of genes of interests were compared with those of the most suitable housekeeping genes, namely GAPDH. The three other tested housekeeping genes (ACTB, OAZ1, and OAZ1) were excluded after analysis of all 5 housekeeping genes using the geNorm software tool^46^ (https://genorm.cmgg.be/). The RT-qPCR efficiencies ranged from 92 to 106% and are listed in Supplementary Table S2. The relative amount of each studied mRNA was normalized to GAPDH mRNA levels as a housekeeping gene, and the data were analysed according to the 2^−△△CT^ method.

### Mucosal microbial DNA extraction, PCR amplification, Illumina MiSeq sequencing and sequencing data processing

One gram of wet colonic mucosa was used for DNA extraction. The DNA was extracted by a bead-beating method using a QIAamp DNA Stool Mini Kit (Qiagen,, Hilden, Germany) according to the manufacturer’s instructions. Briefly, colonic mucosa samples were disrupted in an ASL buffer and homogenised with 100 mg of zirconium beads (0.1 mm) in a Mini-Beadbeater-1 (Biospec Products Inc.) at a rate of 4800 rpm/min four times for 30 s each time at room temperature. Lysozyme was then added at a final concentration of 20 mg/mL (Sigma), and the suspension was incubated at 37 °C for 40 min to improve lysis efficiency. Subsequently, the mixture was incubated in a 95 °C water bath for 5 min to further increase the amount of total DNA extraction. The DNA concentration was measured using a NanoDrop 2000 Spectrophotometer (Thermo-Fisher Scientific). The V3–V4 region of the bacteria 16S rRNA gene were amplified by PCR (95 °C for 2 min, followed by 25 cycles at 95 °C for 30 s, 55 °C for 30 s, and 72 °C for 30 s and a final extension at 72 °C for 5 min) using primers 338F (5′-barcode- ACTCCTRCGGGAGGCAGCAG)-3′ and 806R (5′-GGACTACCVGGGTATCTAAT-3′) (amplicon length: ~ 470 bp), where the barcode was an eight-base sequence unique to each sample. Amplicons were extracted from 2% agarose gels and purified using the AxyPrep DNA Gel Extraction Kit (Axygen Biosciences, Union City, CA, USA) according to the manufacturer’s instructions and quantified using QuantiFluor™ -ST (Promega, USA). PCR production from each sample was used to construct a sequencing library with an Illumina TruSeq DNA Sample Preparation Kit according to the manufacturer’s instruction. Illumina TruSeq PE Cluster and SBS Kit were used to perform cluster generation, template hybridisation, isothermal amplification, linearisation, blocking and denaturisation and hybridisation of the sequencing primers. Paired-end sequencing 2 × 250 bp was performed to sequence all libraries on an Illumina MiSeq platform according to the standard protocols^47^.

Sequences from the Illumina MiSeq platform were processed using the QIIME (version 1.70) software package^48^. Quality filtering was performed with the following criteria: (i) the 250 bp reads were truncated at any site receiving an average quality score <20 over a 10-bp sliding window, discarding the truncated reads that were shorter than 50 bp; (ii) exact barcode matching, two- nucleotide mismatch in primer matching, and reads containing ambiguous characters were removed; (iii) only sequences that overlapped longer than 10 bp were assembled according to their overlap sequence. Reads that could not be assembled were discarded. OTUs were clustered with a 97% similarity cutoff using UPARSE (version 7.1 http://drive5.com/uparse/), and chimeric sequences were identified and removed using UCHIME^49^. The most abundant sequences within each OTU were designated ‘representative sequences’, and 2,203 representative sequence were then aligned against the core set of Greengenes 13.5^50^ using PyNAST^51^ with the default parameters set by QIIME. A PH Lane mask supplied by QIIME was used to remove the hypervariable regions from the aligned sequences. FASTTREE^52^ was used to create a phylogenetic tree of the representative sequences. Sequences were classified using the Ribosomal Database Project classifier with a standard minimum support threshold of 80%^53^. Sequences identified as chloroplasts or mitochondria were removed from the analysis. Community diversity was estimated using the ACE, Chao1, and Shannon indices. The unweighted UniFrac distance method was used to perform a principal coordinate analysis^54^, and an unweighted distance-based analysis of molecular variance was conducted to assess the significant differences among the samples using the MOTHUR programme (version 1.29)^55^.

The 16S sequencing data for all the samples analysed in this study were submitted to the Sequence Read Archive (SRA; http://www.ncbi.nlm.nih.gov/Traces/sra/) under accession SRR2895160.

### Metagenome prediction using PICRUSt

In the present study, we used PICRUSt^56^ to predict the metagenome of each sample. PICRUSt is a bioinformatics tool that uses marker genes, in this case 16S rRNA, to predict the metagenome gene’s functional content. These pre-dictions are precalculated for genes in databases including KEGG^57^ and COGs. In the present study, we used the KEGG database, and closed reference OTU picking was performed using the sampled reads against a Green Genes reference taxonomy (Greengenes 13.5) using the pick_closed_reference_OTU.py script in QIIME^48^. The 16S copy number was normalised using the normalize_by_copy_number.py script, the metagenome functions were predicted using the predict_metagenomes.py and the data were summarised into KEGG pathways using the categorize_by_function.py script, all included in PICRUSt^56^. PICRUSt also calculated the nearest sequenced taxon index (NSTI), a measure of prediction uncertainty presented here in a comparative way along the sequential batches in both consortia datasets. Similarities among bacterial functions were determined using a PCA with the SIMCA-P (11.5) software package (Umetrics, Umea, Sweden).

### Statistical analyses

The statistical calculations were carried out with tests using the SPSS software package (SPSS v. 16, SPSS Inc., Chicago, IL, USA). The goat was the experimental unit for all comparisons, and diet was regarded as the fixed effect. The normality of the distribution of variables was tested by the Shapiro–Wilk test. The independent samples T-test procedure was used to analyse the variables found to have a normal distribution. The variables found to have a non-normal distribution were analysed using the Kruskal–Wallis test procedure. Significance was declared at *P* < 0.05.

Double dendograms were constructed using the comparative functions and multivariate hierarchical clustering methods of Hemi^58^, on the basis of the abundances of the bacterial groups at different taxonomic levels. Correlations between colonic pH, colonic LPS and the mucosa-associated microbiota, and inflammatory cytokine expression were assessed by Pearson’s correlation test using GraphPad Prism version 6.00 (GraphPad Software, San Diego, CA, USA). Significance was declared at *P* < 0.05.

## Additional Information

**How to cite this article**: Ye, H. *et al.* Grain-rich diets altered the colonic fermentation and mucosa-associated bacterial communities and induced mucosal injuries in goats. *Sci. Rep.* **6**, 20329; doi: 10.1038/srep20329 (2016).

## Supplementary Material

Supplementary Information

## Figures and Tables

**Table 1:** Effects of high-grain (HG) feeding on colonic digesta variables in goats at the time of slaughter (n = 6).

Items	Hay	HG	SEM^a^	*P*-value
Colonic pH	7.44	6.66	0.138	0.003
Acetate (μmol/g)	25.65	60.11	5.400	<0.001
Propionate (μmol/g)	5.17	12.07	1.123	<0.001
Isobutyrate (μmol/g)	0.86	1.63	0.131	<0.001
Butyrate (μmol/g)	1.31	5.14	0.701	0.005
Isovalerate (μmol/g)	0.77	1.50	0.141	0.003
Valerate (μmol/g)	0.49	1.11	0.118	0.006
Total SCFA (μmol/g)	34.25	81.56	7.428	<0.001
Lactate (μmol/g)	1.78	3.12	0.215	<0.001
LPS (EU/g)	12066	39258	5506	0.011

^a^SEM, Standard Error of the Mean.

**Table 2:** Effects of high-grain (HG) diet feeding on the average richness and diversity of colonic mucosal bacterial community at the 3% dissimilarity level (n = 6).

Items	Hay	HG	SEM^a^	*P*-value
OTUs^b^	1056	815	44	0.001
ACE^c^	1117	861	47	0.001
Chao 1	1136	838	49	0.001
Shannon index	4.68	4.57	0.074	0.513

^a^SEM, Standard Error of the Mean.

^b^OTU, operational taxonomic units.

^c^ACE, abundance-based coverage estimator.

**Table 3:** Effects of high-grain (HG) feeding on average relative abundance of genus level (% of total sequences) in colonic mucosa, ranked by alphabetical order of first letter of phylum, family and genus name (n = 6).

Phylum	Family	Genus/Other	Abundance (%)^a^		SEM^b^	*P*-value
			Hay	HG		
Bacteroidetes	Bacteroidaceae	*Bacteroides*	1.13	1.65	0.298	0.411
	Rikenellaceae	*Alistipes*	0.92	2.04	0.309	0.086
		Unclassified Rikenellaceae	2.82	3.39	0.543	0.626
	Ruminococcaceae	Unclassified Ruminococcaceae	14.69	20.58	1.568	0.055
	Unclassified Bacteroidales	Unclassified Bacteroidales	1.98	2.90	0.419	0.291
Firmicutes	Bacillaceae	*Bacillus*	1.01	0.69	0.080	0.045
	Christensenellaceae	Unclassified Christensenellaceae	3.39	2.40	0.291	0.087
	Enterococcaceae	*Enterococcus*	3.88	2.64	0.284	0.021
	Lachnospiraceae	*Blautia*	0.07	1.21	0.249	0.029
		Unclassified Lachnospiraceae	1.67	2.60	0.253	0.073
	Lactobacillaceae	*Lactobacillus*	1.26	0.69	0.400	0.933
	Streptococcaceae	*Lactococcus*	28.88	19.17	2.380	0.033
	Peptostreptococcaceae	Unclassified Peptostreptococcaceae	2.54	0.77	0.337	0.002
	Prevotellaceae	*Prevotella*	0.15	4.44	1.194	0.098
	Unclassified Clostridiales	Unclassified Clostridiales	3.45	2.24	0.379	0.111
Proteobacteria	Comamonadaceae	*Aquabacterium*	1.09	0.32	0.460	0.384
	Moraxellaceae	*Psychrobacter*	1.01	0.63	0.080	0.013
	Pseudomonadaceae	*Pseudomonas*	6.37	4.10	0.562	0.036
Spirochaetae	Spirochaetaceae	*Treponema*	3.29	2.24	1.011	0.627
Unclassified bacteria	Unclassified bacteria	Unclassified bacteria	1.12	2.81	0.550	0.152

^a^Only results obtained for the predominant bacterial genera (average relative abundance >1% in at least one group) are presented.

^b^SEM, Standard Error of the Mean.
