# Supplementary material for: Grain-rich diets altered the colonic fermentation and mucosa-associated bacterial communities and induced mucosal injuries in goats
Source: Sci Rep. 2016 Feb 4;6:20329. doi: 10.1038/srep20329 (PMC4740883; doi:10.1038/srep20329)
Supplement: Supplementary Information [file srep20329-s1.doc]

**Supplementary Figures and Tables**

**Grain-rich diets altered the colonic fermentation and mucosa-associated bacterial communities and induced mucosal injuries in goats**

Huimin Ye, Junhua Liu, Panfei Feng, Weiyun Zhu, Shengyong Mao *

College of Animal Science and Technology, Nanjing Agricultural University, Nanjing 210095,China*

**Supplementary Figure S1**

**Supplementary Figure S2**

**Supplementary Figure S3**

**Supplementary Figure S4**

**Supplementary Figure S5**

**Supplementary Figure S6**

**Supplementary Table S1**

**Supplementary Table S2**

Figure S1. The relative abundance of phylum in the composition of colon mucosa-associated microbiota of goats. HG: high-grain.

Figure S2. Influence of feeding high-grain (HG) diets on the top 50 most abundant genera.


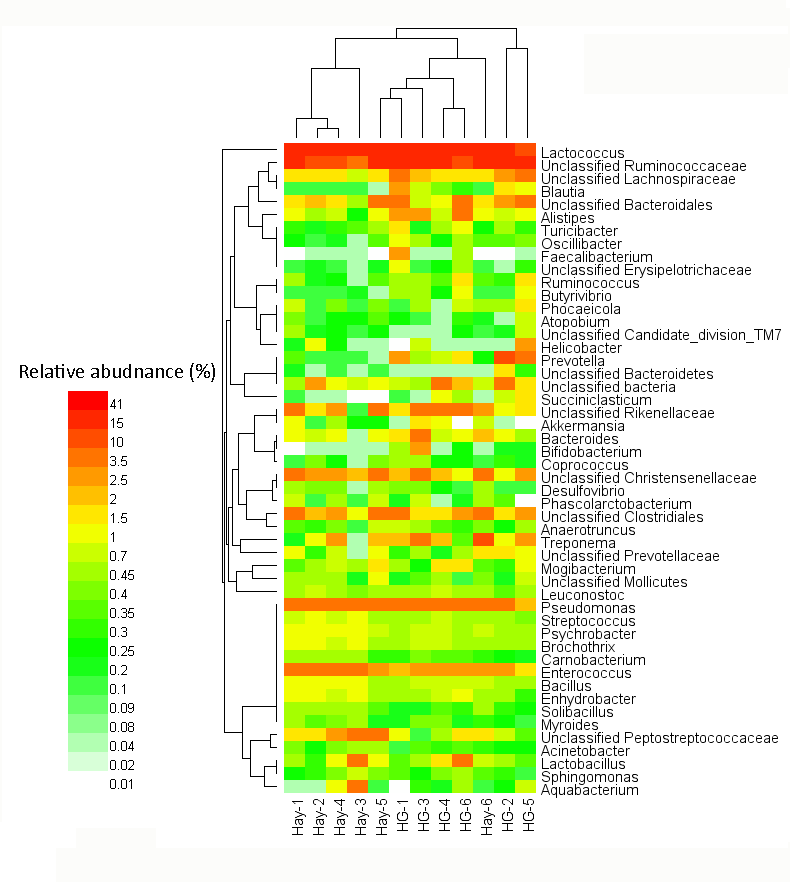


Figure S3. Summary of rarefaction results based on operational taxonomic unit (OTUs) (3% divergence) for each sample. Hay group: Hay-1, Hay-2, Hay-3, Hay-4, Hay-5, Hay-6; High grain group: HG-1, HG-2, HG-3, HG-4, HG-5, HG-6.

Figure S4. Venn diagram of shared OTUs between the hay and high-grain (HG) group microbiomes.


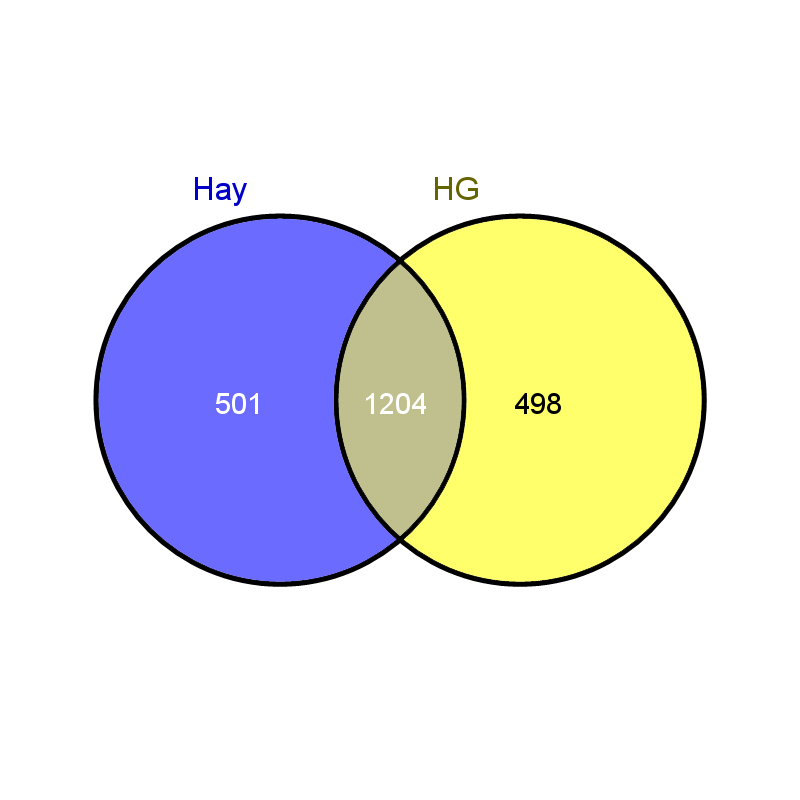


Figure S5. Comparison of the dominant operational taxonomic units (OTU) (relative abundance ≥1% in at least one group) in colonic mucosal samples of goats. S:=species; G: =genus; F: =family.

Figure S6. Relative abundances of metabolic pathways encoded in the microbiota of colonic mucosa in hay and high-grain (HG) group.

Table S1. Ingredient and nutrient levels of the experimental diets

| Items | Hay HG | | |
| --- | --- | --- | --- |
| Ingredients (% of DM) |  | | |
| Leymus chinensis | 80.00 | 18.00 | |
| Medicago sativa hay | 16.00 | 7.00 | |
| Corn | 0.00 | 20.00 | |
| Wheat | 0.00 | 36.50 | |
| Soybean meal | 0.00 | 15.00 | |
| Limestone meal | 0.70 | 1.00 | |
| Calcium phosphate dibasic | 1.80 | 1.00 | |
| Salt | 0.50 | 0.50 | |
| Premix | 1.00 | 1.00 | |
| Total | 100.00 | 100.00 | |
| Nutrient levels |  | | |
| Net energy (MJ/kg) | 8.32 | | 11.56 |
| Crude protein (%) | 10.16 | | 17.19 |
| Neutral detergent fiber (%) | 56.84 | | 22.75 |
| Acid detergent fiber (%) | 35.70 | | 12.78 |
| Calcium (%) | 1.24 | | 0.82 |
| Phosphorus (%) | 0.50 | | 0.55 |

Table S2. Primers used for quantitative real-time PCR analysis

| Gene Name | Forward and Reverse Sequences | Reference | Amplicon Size, bp | Effeciency, % |
| --- | --- | --- | --- | --- |
| Claudin-1 | F: CACCCTTGGCATGAAGTGTA | Liu *et al*., 2013 | 216 | 102 |
|  | R: AGCCAATGAAGAGAGCCTGA |  |  |  |
| Claudin-4 | F: AAGGTGTACGACTCGCTGCT | Liu *et al*., 2013 | 238 | 103 |
|  | R: GACGTTGTTAGCCGTCCAG |  |  |  |
| Claudin-7 | F: AGACGACAAAGTGAAGAAGG | Liu *et al*., 2013 | 298 | 105 |
|  | R: CTTGGAAGAGTTGGACTTAGG |  |  |  |
| Occludin | F: GTTCGACCAATGCTCTCTCAG | Liu *et al*., 2013 | 200 | 93 |
|  | R: CAGCTCCCATTAAGGTTCCA |  |  |  |
| ZO-1 | F: CGACCAGATCCTCAGGGTAA | Liu *et al*., 2013 | 163 | 95 |
|  | R: AATCACCCACATCGGATTCT |  |  |  |
| IL-1β | F: CATGTGTGCTGAAGGCTCTC | Liu *et al*., 2013 | 173 | 104 |
|  | R: AGTGTCGGCGTATCACCTTT |  |  |  |
| IL-2 | F: AGTCATTGCTGCTGGATTTACA | Liu *et al*., 2013 | 222 | 106 |
|  | R: CCATTGAATCCTTGATCTCTCTG |  |  |  |
| IL-6 | F: CCAATCTGGGTTCAATCAGG | Liu *et al*., 2013 | 241 | 103 |
|  | R: ACCCACTCGTTTGAGGACTG |  |  |  |
| IL-10 | F: TTAAGGGTTACCTGGGTTGC | Liu *et al*., 2013 | 239 | 92 |
|  | R: CCCTCTCTTGGAGCATATTGA |  |  |  |
| IL-12 | F: GGATCAGAAAGAACCCAAAGC | Liu *et al*., 2013 | 208 | 106 |
|  | R: ATACTCCCTGTGGTCCATGC |  |  |  |
| TNF-α | F: CAAGTAACAAGCCGGTAGCC | Liu *et al*., 2013 | 155 | 105 |
|  | R: AGATGAGGTAAAGCCCGTCA |  |  |  |
| IFN-γ | F: TGATTCAAATTCCGGTGGAT | Liu *et al*., 2013 | 166 | 102 |
|  | R: GCAGGCAGGAGAACCATTAC |  |  |  |
| GADPH | F: GGGTCATCATCTCTGCACCT | Wang *et al*., 2012 | 180 | 98 |
|  | R: GGTCATAAGTCCCTCCACGA |  |  |  |

Liu, J. H., Xu, T. T., Liu, Y. J., Zhu, W. Y., & Mao, S. Y. (2013). A high-grain diet causes massive disruption of ruminal epithelial tight junctions in goats.*American Journal of Physiology-Regulatory, Integrative and Comparative Physiology*, *305*(3), R232-R241.

Wang, A., Akers, R. M., & Jiang, H. (2012). Short communication: Presence of G protein-coupled receptor 43 in rumen epithelium but not in the islets of Langerhans in cattle. *Journal of dairy science*, *95*(3), 1371-1375.
